# A comparison of machine learning algorithms in predicting COVID-19 prognostics

**DOI:** 10.1007/s11739-022-03101-x

**Published:** 2022-09-18

**Authors:** Serpil Ustebay, Abdurrahman Sarmis, Gulsum Kubra Kaya, Mark Sujan

**Affiliations:** 1 Department of Computer Engineering, Istanbul Medeniyet University, Istanbul, Turkey; 2 Department of Microbiology Laboratory, Goztepe Prof. Dr. Suleyman Yalcin City Hospital, Istanbul, Turkey; 3 Department of Industrial Engineering, Istanbul Medeniyet University, Istanbul, Turkey; 4 School of Aerospace, Transport and Manufacturing, Cranfield University, Bedford, MK430AL UK; 5 Human Factors Everywhere, Woking, UK

**Keywords:** COVID-19, Infectious diseases, Machine learning, Prognostic predictions, Risk factors

## Abstract

ML algorithms are used to develop prognostic and diagnostic models and so to support clinical decision-making. This study uses eight supervised ML algorithms to predict the need for intensive care, intubation, and mortality risk for COVID-19 patients. The study uses two datasets: (1) patient demographics and clinical data (*n* = 11,712), and (2) patient demographics, clinical data, and blood test results (*n* = 602) for developing the prediction models, understanding the most significant features, and comparing the performances of eight different ML algorithms. Experimental findings showed that all prognostic prediction models reported an AUROC value of over 0.92, in which extra tree and CatBoost classifiers were often outperformed (AUROC over 0.94). The findings revealed that the features of C-reactive protein, the ratio of lymphocytes, lactic acid, and serum calcium have a substantial impact on COVID-19 prognostic predictions. This study provides evidence of the value of tree-based supervised ML algorithms for predicting prognosis in health care.

**Supplementary Information:**

The online version contains supplementary material available at 10.1007/s11739-022-03101-x.

## Introduction

The COVID-19 pandemic resulted in intense pressure on healthcare providers worldwide, especially in low- and middle-income countries (LMICs), where resources are limited [1–4]. At the time of writing this article, the virus had spread worldwide with over 596 million cases leading to over 6.4 million deaths in 190 countries [5].

It has been established that patients with COVID-19 may experience worsening conditions a few days after contracting the infection [6, 7]. The time estimated from the disease onset to Intensive Care Unit (ICU) admission is between 9 and 12 days [8, 9]. Similarly, the approximate length of a patient’s stay in the ICU is 9 days [10, 11]. Considering the fact that approximately 26–32 percent of hospitalized COVID-19 patients are admitted to the ICU, increased hospital resources are required during the pandemic (e.g., healthcare staff, hospital beds, and mechanical ventilators) [12, 13]. Although the global vaccination program eased the pressure on healthcare providers, not all countries had equal access to vaccine products [14]. In this respect, developing diagnostic and prognostic models becomes a valuable contribution [12].

Machine Learning (ML) algorithms have supported clinical decision-making [15–18]. ML algorithms are built on statistics and used in healthcare to diagnose diseases and develop prognostic models. For example, Glotsos et al. [19] used Support Vector Machines (SVM) to assist in diagnosing brain tumor astrocytoma. Scioscia et al. [20] used SVM to predict continuous positive airway pressure. Garcia Carretero et al. [21] used multiple ML algorithms to predict vitamin D deficiency in a hypertensive population.

So far, ML has been used to develop COVID-19 diagnostic and prognostic predictive models using computed tomography (CT) images, laboratory blood test results, and patient comorbidities [22–24]. For example, Ismael and Sengur [22] used 95 chest X-ray images to detect COVID-19. They reached an accuracy rate of 0.947 using the ResNet50 model and support vector machine (with the linear kernel) classifier. Despite the high accuracy rate obtained from computed tomography (CT) images, several researchers suggested using laboratory blood tests and clinical measurements as they are more accessible and less expensive [3, 25]. Blood tests and clinical measurements are routinely collected in high and middle-income countries [25].

Alakus and Turkoglu [26] developed a model to predict COVID-19 diagnosis using laboratory blood test features from 600 patients. They reached an accuracy rate of 0.866 using a deep learning algorithm of long–short-term memory. Yan et al. [13] predicted mortality risk levels using 485 COVID-19 patients’ data. The study used the extreme gradient-boosting (XGBoost) algorithm and performed with over 0.90 accuracy. What is more, some studies primarily used clinical data. Despite the availability of ML studies, those studies often developed a single prediction model such as diagnosis, mortality risk, and need for intubation using a small dataset.

This study uses eight supervised ML algorithms to predict COVID-19 prognostics using 11,712 observations from hospitalized COVID-19 patients in Turkey. The study has three aims: (1) to develop prediction models for the need for intensive care, the need for intubation, and the risk of mortality, (2) to identify the importance of clinical and blood test features in each prognostic prediction model, and (3) to compare the performances of eight supervised ML algorithms that were developed using different approaches, namely regression-based (i.e., logistic regression), margin-based (i.e., support vector machine), artificial neural network-based (i.e., MLP), ensemble-based (i.e., random forest, XGBoost, CatBoost, and extra trees), and instance-based (i.e., k-NN).

## Materials and methods

This study is designed by following five steps: data collection, data pre-processing, data analysis, model building, and performance evaluation. Each step is explained in the following sections.

### Data collection

This study collected patient demographics, clinical data (e.g., vitals and chronic diseases), and laboratory blood tests.

Patient demographics included two features: age and gender. Seventeen features were collected as part of clinical data, namely: temperature, heart rate, oxygen saturation, blood pressure, pupils, consciousness, general condition, diuresis, cardiovascular diseases, hypertension, diabetes mellitus, neurological diseases, respiratory diseases, benign prostate hyperplasia, chronic renal failure, hepatitis C, and cancer. Eighteen features were collected for laboratory blood test data: alanine aminotransferase, aspartate aminotransferase, white blood cell, platelet, mean platelet, eosinophil neutrophil, lymphocyte, basophil, lactate dehydrogenase, glucose, urea, albumin, sodium, potassium, magnesium, C-reactive protein, and creatinine.

This study collected the raw data from hospitalized COVID-19 patients admitted to a public teaching hospital in Istanbul, Turkey, between April 2020 and February 2021. The study only collected patient data for subjects over 18 years old due to the limited data available for patients below 18 years old. All data were extracted from the electronic medical record system. The authors obtained ethical approval (ID 2021/0071) from the hospital to collect the data. Patients’ confidentiality was protected under the hospitals’ policies. Data were analyzed after removing all personal identifiers.

### Data pre-processing

**Fig. 1:**
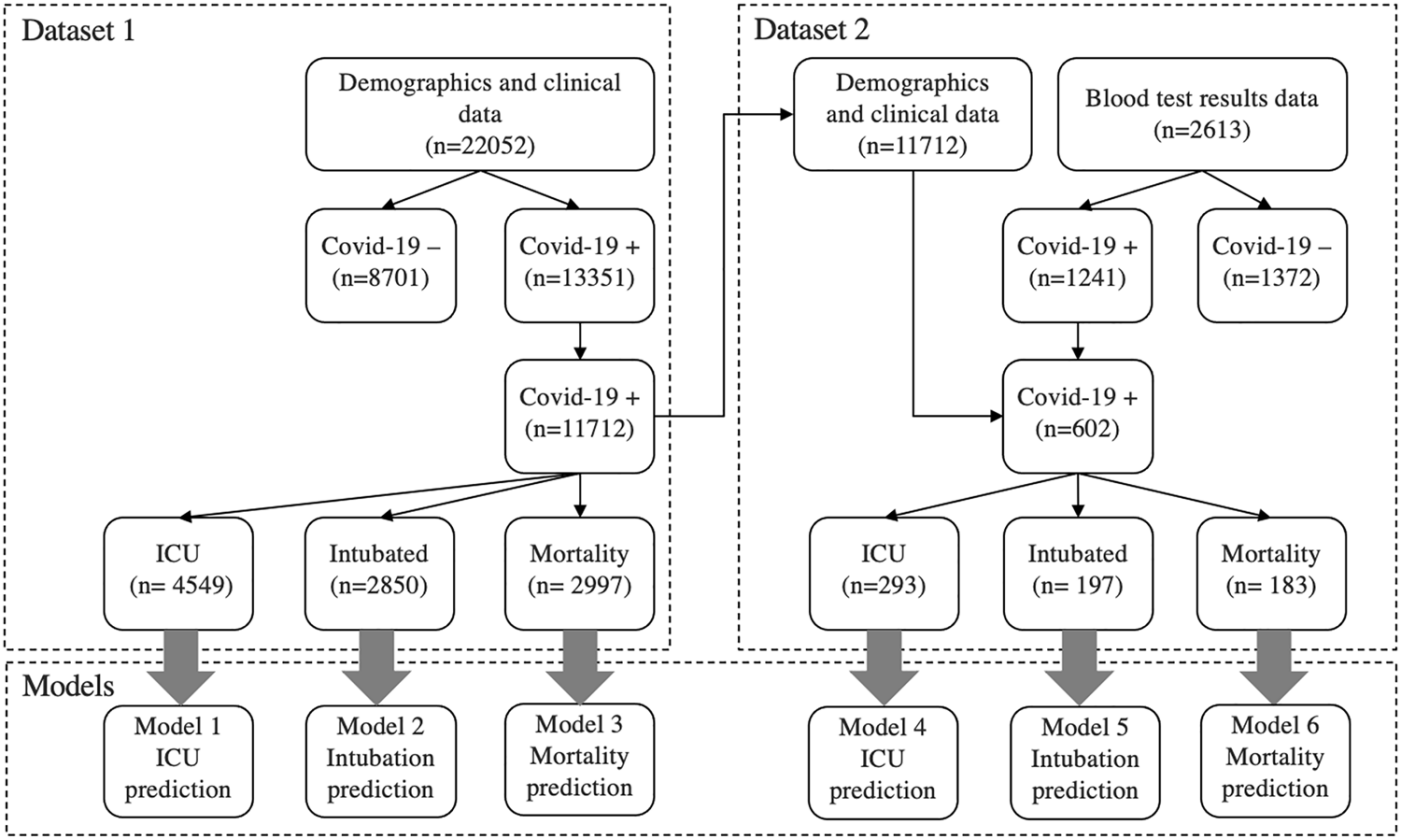
Datasets used to develop the prediction models

The raw data were pre-processed before training the models (Fig. 1). The data pre-processing step involves data cleaning and imputation of the missing values.

This study only included COVID-19 positive cases as the study develops COVID-19 prognostic predictions. The study used two raw datasets for developing the prediction models. Figure 1 shows the number of observations at each dataset collected, after data exclusion, and for each model. The first raw dataset contains inpatients’ demographics and clinical data from 13,351 positive COVID-19 records. The second raw dataset contains inpatients’ demographics and clinical and blood test data from 1241 COVID-19 positive records.

In this study, both datasets contained missing values at specific features. In this matter, the authors made assumptions about the missing values after consulting a healthcare professional. Some records did not include details of chronic diseases, diuresis, or pupils-related features in the first raw dataset. This was due to the healthcare professionals’ record-keeping habits. Not all clinical data were recorded the same way; some healthcare professionals recorded only deteriorating conditions or negative situations. In such cases, the authors consulted a healthcare professional and made assumptions accordingly. The authors assumed that the feature was considered normal if the patient’s clinical data did not mention any of these features. After imputing assumed values, all records with at least one missing value were dropped and not used for further analysis. As a result, Models 1, 2, and 3 were built using 11,712 COVID-19 positive records from 1256 patients.

The authors combined the first dataset with patients’ laboratory blood test data in the second dataset. The authors reviewed the blood test data of 1241 COVID-19 positive records to manage the missing values. Missing values in more than or equal to ten percent of the instances were dropped and not used for further analysis. Missing values were imputed using the same patients’ other blood test results. Each patient’s missing value was supplied by taking the average value of the same patient’s other blood test results. Similar studies often assumed taking the average feature value among all patients’ data [3, 27], potentially leading to more noise and overfitting. The approach taken in this study is clinically more reasonable since a patient’s blood test findings do not change much within a week. However, such an approach would require more time and effort because a healthcare professional’s input is needed for reviewing and confirming each imputed value. After managing the missing values and combining them with the first dataset, Models 4, 5, and 6 were built using 602 positive COVID-19 records from 91 patients.

### Data analysis

Before moving to model training, this step analyzed features in each dataset. This study collected data from a cohort of 5112 women and 6600 men aged between 18 and 97. Supplementary Table S1 provides the descriptive statistics of features from the first dataset, and Supplementary Table S2 provides the descriptive statistics from the second dataset.

Pearson correlation coefficients (*r*) were calculated for each dataset to remove the features having a high correlation (*r* above 0.9) before developing the prediction models [17, 28]. This procedure was also applied in other studies to minimize overfitting. Complex models, having many variables, often experience overfitting [17].

Supplementary Table S3 demonstrates a correlation matrix for demographic and clinical data features. Results showed almost no significant correlation between the features, except consciousness and general condition (*r* = – 0.77). There was also a moderate to weak correlation between the general condition and diastolic blood pressure (*r* = 0.46), consciousness and diastolic blood pressure (*r* = 0.39), and diabetes and hypertension (*r* = 0.36). Consequently, all nineteen features were used to build Models 1, 2, and 3.

Supplementary Table S4 shows the correlation matrix for the features of demographic, clinical data, and blood test results. Stronger correlations were observed between the features in the second dataset. The findings revealed a significant correlation between neutrophil count and white blood cell count (*r* = 0.99). As a result, the feature neutrophil count was removed when building Models 4, 5, and 6.

### Model building

Using each dataset, this study developed three prediction models for hospitalized COVID-19 patients: the need for intensive care, intubation, and mortality risk. In total, six models were developed, each using eight ML algorithms (see Fig. 1).

Each patient received a value between 0 and 1 for each prognostic prediction model. As multiple prediction models were developed for each patient, this study proposes a different application to address multi-label classification problems. Support vector machines (with the linear kernel), logistic regression, random forest, XGBoost, multilayer perceptron, extra trees, CatBoost, and k-nearest neighbors classifiers were used to develop the prediction models.

This study calculated XGBoost feature importance values to determine the individual contribution of each feature to the prognostic predictions. XGBoost is a learning framework that is based on boosting tree models. It is generalizable and achieves better performance in practical applications [29].

### Machine learning algorithms

ML algorithms can reveal the complex non-linear relationships between the input and output data. The authors selected algorithms based on their fundamental ML task types and their strengths and weaknesses. For instance, SVM supports linear and non-linear solutions, whereas logistic regression can only work with linear ones. The MLP algorithm has a complex architecture to define relationships between data; however, it is difficult to explain the learning phase. In contrast, a decision tree makes a complex predictive model much easier to interpret by representing the findings visually. Ensemble-based algorithms combine forecasts from multiple models. That can reduce variance and minimize bias. The *k*-NN algorithm has non-parametric architecture, simple and powerful. All these algorithms performed well in other studies.

Support vector machines (SVM) find a line representing the best fit between two output classes [30]. The mathematical expression of the SVM-linear kernel is shown in Eq. (1), where ${c}_{i}$ is obtained by solving the optimization problem, which can be solved by quadratic programming, ${y}_{i}$ is the class (0 or 1) of the value ${x}_{i}$, and ${\varphi (x}_{i})$ is the transformed data point [31].

1 $$f\left(x\right)=\sum_{i=1}^{n}{c}_{i}{y}_{i}\varphi ({x}_{i})$$

Logistic regression (LR) uses Eq. (2), where *b*0 and *b*1 are learned by training the data [32]. The algorithm aims to minimize the error between the predicted and actual outcomes. LR is prone to overfitting, which could be overcome by removing the highly correlated features [17].

2 $$P\left(x\right)={e}^{b0+b1x}/(1+{e}^{b0+b1x})$$

Random forest (RF) is developed to overcome the risk of overfitting in decision trees, and RF is an example of bagging, an ensemble technique [33]. It works well when limited data are available. RF handles the missing values [17]. The output function is obtained as in Eq. (3), where ${p}_{t}(y/x)$ is the probability distribution of each tree (*t*), and *x* is the set of test samples [34].

3 $$Z=argmax \frac{1}{T}\sum_{t=1}^{T}{p}_{t}(y/x)$$

Extreme gradient boosting (XGBoost) is similar to the random forest but is an example of boosting. At each tree, previously incorrectly classified data are trained. Gradient boosting is made from weak predictors [35]. The output function, $F\left({x}_{i}\right)$, is obtained by Eq. (4), where ${x}_{i}$ is the explanatory variable, and ${F}_{t}({x}_{i})$ is the output function of each tree [29].

4 $$F({x}_{i})=\sum_{t=1}^{T}{F}_{t}({x}_{i})$$

Multilayer perceptron (MLP) is an artificial neural network, which is composed of an input, a hidden, and an output layer(s) [36]. The numbers of hidden layers are calculated by trial and error. The basic mathematical illustration for the output is shown in Eq. (5), where j represents the neuron, ${f}_{i}$ is any non-linear function, ${x}_{i}$ is the input signal, and ${w}_{ji}$ is the weights [37].

5 $${y}_{i}={f}_{i}\left(\sum_{i}{w}_{ji} {x}_{i}\right)$$

Extra trees (ET) are an ensemble machine learning algorithm that combines the predictions of many decision trees. ET was developed on random forest trees and is less prone to the risk of overfitting [38, 39]. The main difference lies in the selection of cut points to split nodes. The random forest chooses the optimum split, whereas ET chooses it randomly.

CatBoost is a recently developed gradient-boosting algorithm [40]. Binary decision trees are used for the base predictor. Equation (6) shows the estimated output description, where $H\left({x}_{i}\right)$ is the decision tree function, ${x}_{i}$ is the explanatory variable and ${R}_{j}$ is the disjoint region. CatBoost differs from other gradient-boosting algorithms in three points; it uses ordered boosting, it can be used with small data size, and it can handle categorical features [34].

6 $$H({x}_{i})=\sum_{j=1}^{J}{c}_{j}{1}_{\left\{x\epsilon {R}_{j}\right\}}$$

*K*-nearest neighbors classifiers (*k*-NN) is a non-parametric technique that labels an unknown object in the same class as the majority of the *k*-nearest neighbors [41]. In so doing, the Euclidean distance between the unknown object and its neighbor is calculated as in Eq. (7). It might not be ideal to use *k*-NN with high dimensional data as it requires extensive computing efforts [17].

7 $$d\left(A, B\right)=\sqrt{\sum_{i=1}^{n}({{a}_{i-}{b}_{i})}^{2}}$$

### Performance evaluation

The performance of each prediction model was assessed in terms of positive predictive value, negative predictive value, positive likelihood ratio, negative likelihood ratio, accuracy, sensitivity, specificity, *F*1 score, and the area under the ROC curve (AUROC). Model calibration was made using Platt scaling method with sigmoid regression. We measured calibration with a scaled Brier score, which goes beyond traditional calibration and discrimination measures by evaluating the clinical usability of models [42].

## Experimental results

This study developed six models for the three prognostic predictions (i.e., the need for intensive care, the need for intubation, and the risk of mortality) using two datasets: (1) patient demographics and clinical data (*n* = 11,712) and (2) demographics, clinical data, and blood test results (*n* = 602). The datasets were randomly split; 70 percent of the data was used for training, and 30 percent was for testing. The tenfold cross-validation procedure was used for estimating the performance of ML algorithms. The hyperparameters have been optimized by defining a grid of hyperparameter ranges using the Scikit-Learn Randomized Search CV method. It randomly samples from the grid and performs cross-validation with each tested value combination [43]. Supplementary Table S5 shows the hyperparameters used in this study.

This study used Python 3.6 to develop all models, evaluate model performances and undertake statistical analysis. The study used the codes provided by Fernandes, F.T. et al. [3] to create the prediction models. All tests were executed on an Intel Core I5 computer based on Windows 10 OS.

### Model performance

The best algorithm for each model was selected based on AUROC values due to its strong ability to distinguish between positive and negative classes (Table 1). Model calibration of the best algorithm was measured using a scaled Brier score, where the perfect model achieves a score of 1 (Table 2).

**Table 1:** Comparison of algorithm performances

ML algorithms	Model 1 AUROC	Model 2 AUROC	Model 3 AUROC	Model 4 AUROC	Model 5 AUROC	Model 6 AUROC
Extreme gradient boosting	**0.926**	0.959	0.944	0.975	0.940	0.978
CatBoost classifier	0.924	**0.961**	**0.946**	0.977	0.945	0.984
Extra Trees classifier	0.904	0.961	0.939	**0.989**	**0.947**	**0.990**
Random forest classifier	0.864	0.953	0.908	0.945	0.925	0.978
MLP classifier	0.859	0.959	0.922	0.977	0.930	0.983
Logistic regression	0.811	0.957	0.878	0.864	0.942	0.949
Support vector machine- linear kernel	0.808	0.956	0.874	0.876	0.932	0.943
*K* neighbors classifier	0.848	0.945	0.890	0.904	0.912	0.932

The bold numbers are the AUROC values that received the highest score in each model.

**Table 2:** Model performance for the best-performed algorithm

Prediction model	Best algorithm	Model performance									
		Accuracy	AUROC (95% CI)	Sensitivity	Specificity	PPV	NPV	F1	PLR	NLR	Scaled brier
Model 1	XGBoost	0.75	0.90 (0.89, 0.91)	0.899	0.657	0.621	0.913	0.74	2.63	0.15	0.297
Model 2	CatBoost	0.915	0.96 (0.95, 0.97)	0.918	0.913	0.754	0.975	0.83	10.63	0.09	0.609
Model 3	CatBoost	0.86	0.946 (0.94, 0.95)	0.886	0.857	0.662	0.959	0.76	6.22	0.13	0.462
Model 4	ET	0.95	0.987 (0.98, 1)	0.965	0.946	0.944	0.967	0.95	17.96	0.04	0.754
Model 5	ET	0.92	0.973 (0.95, 1)	0.881	0.943	0.881	0.942	0.88	15.36	0.13	0.696
Model 6	ET	0.95	0.993 (0.99, 1)	0.945	0.952	0.896	0.976	0.92	19.85	0.06	0.75

The findings showed that all seven algorithms outperformed SVM in Models 1 and 3. Additionally, all seven algorithms outperformed the *k*-NN algorithm in Models 2, 5, and 6, and the LR algorithm in Model 5.

Table 2 shows performance evaluation findings from the highest performed algorithm. All models reported a high predictive performance on the test data with an AUROC value of over 92 percent, which shows the discriminative ability of models.

### Feature importance

**Fig. 2:**
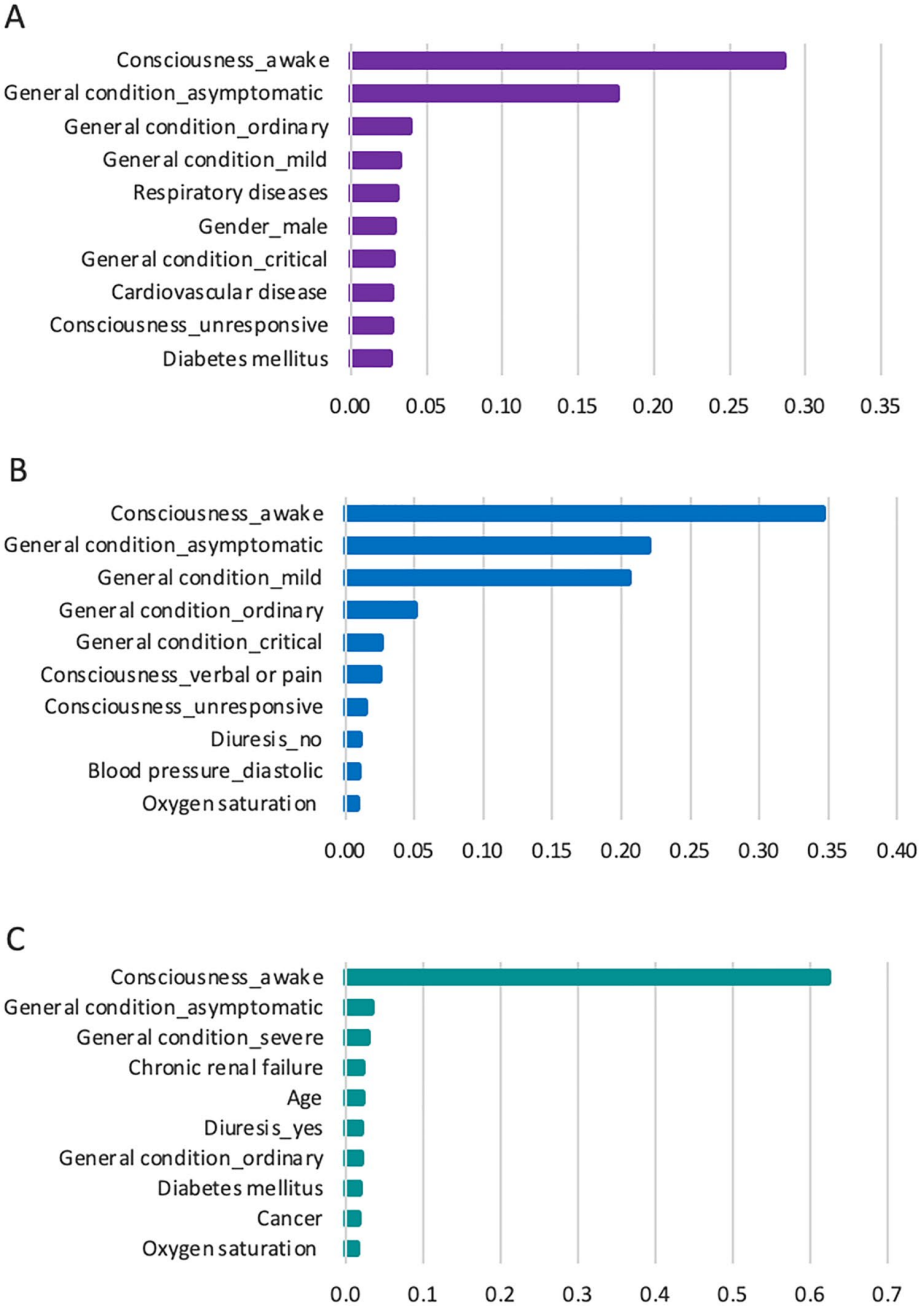
Feature importance for predicting models. **A** Feature importance in Model 1 (the need for intensive care), **B** Feature importance in Model 2 (the need for intubation), and **C** Feature importance in Model 3 (the risk of mortality)

XGBoost feature importance values were calculated to analyze the importance of each feature on the prognostic prediction of each model. Figure 2 illustrates the top ten critical features from demographics and clinical data for predicting the need for intensive care (Model 1), the need for intubation (Model 2), and the risk of mortality (Model 3). These features have the highest impact on predictions. The complete list of feature importance values is shown in Supplementary Figs. S1, S2, and S3. The study found that consciousness and general condition have the highest impact on predicting the prognosis of COVID-19. For example, respiratory or cardiovascular disease appears to have a substantial effect on predicting patients needing ICU, and oxygen saturation is a critical feature in predicting the need for intubation and the risk of mortality.

**Fig. 3:**
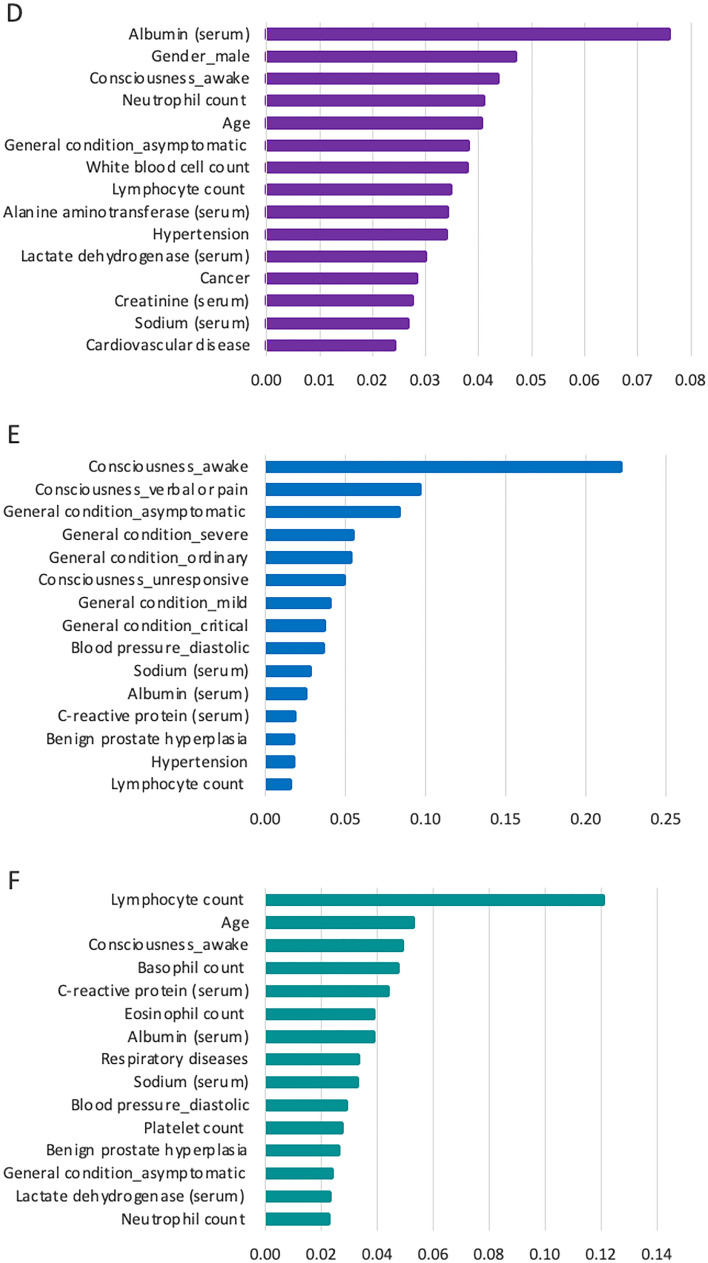
Feature importance for predicting models. **D** Feature importance in Model 4 (the need for intensive care), **E** Feature importance in Model 5 (the need for intubation), and **F** Feature importance in Model 6 (the risk of mortality)

Similarly, Fig. 3 shows the top fifteen critical features for predicting Models 4, 5, and 6. The complete list of the feature importance is provided in Supplementary Figs. S4, S5, and S6. For example, the findings showed that serum albumin level has the highest impact on predicting the need for intensive care, and lymphocyte count has the highest impact on predicting mortality risk.

## Discussion

This study used SVM, LR, RF, XGBoost, MLP, ET, CatBoost, and k-NN classifiers to develop COVID-19 prognostic prediction models. The findings revealed that tree-based classifiers (e.g., CatBoost, XGBoost, and ET), as a component of ensemble models, provide a higher level of AUROC for predicting COVID-19 prognostics than regression, margin, neural network, and instance-based algorithms. One reason could be that tree-based classifiers better expose the non-linear relationships by partitioning training data into subsets. Moreover, empirical and theoretical evidence shows that ensemble techniques act as variance reduction mechanisms; they reduce the variance component of the error [44]. Several other studies also provided evidence of cases where ensemble-based models perform better than other ML algorithms [45–47].

SVM is a well-known and widely preferred algorithm in ML [22, 48]. However, despite its popularity, this study received poor algorithm performance from using SVM and k-NN. Nevertheless, contradictory findings are also available in the literature, such as SVM and LR algorithms outperforming RF and XGBoost algorithms [49] and SVM outperforming MLP and RF algorithms [50]. Given the findings of this study, the authors suggest using tree-based classifiers instead of SVM in prognostic predictions, especially when the models are built on large datasets with many features. Both SVM and k-NN algorithms require too much training time, and so incur a higher computational cost [17].

Considering the performances reported in other studies, literature review findings showed an accuracy rate above 0.80, with a few studies reaching 0.99 [45, 51]. This study reports one of the highest performances in predicting the need for intensive care and mortality risk. However, it should be noted that such metrics might not reflect the applicability of these findings or their use in clinical practices [42].

This study calculated XGBoost feature importance values to understand the role of each feature in predicting COVID-19 prognostics. The study provided significant findings, especially in blood test-related features. On the one hand, this study obtained results consistent with those of other studies by recognizing the features of C-reactive protein, the ratio of lymphocytes, lactic acid, and serum calcium as having a substantial impact on COVID-19 prognostic predictions [3, 12, 52, 53]. On the other hand, this study provided further evidence of basophil count, eosinophil count, and serum albumin level on COVID-19 prognostic predictions.

In this study, lymphocytes are the most critical feature in predicting mortality (Fig. 3). Lower lymphocyte counts and higher neutrophil counts impact the prediction of a worse prognosis (Supplementary Figs. S4 and S6). Clinical findings showed that a lower level of blood lymphocyte count is associated with mortality and deteriorating conditions in COVID-19 patients [54–56]. Similarly, our study provided evidence of the significance of basophils and eosinophils when predicting mortality, which is compatible with the results from other studies [25, 51, 57, 58]. Hence, it is reasonable to say that taking routine blood test samples and examining the complete blood test results would facilitate reliable prognostic predictions.

Serum albumin level was found to be another significant feature. It has been widely used as a negative acute-phase reactant, which means a decrease in its level indicates acute inflammation. A reduction in serum albumin levels increases the risk of mortality [59, 60]. Kheir et al. [61] reported the mean serum albumin level for the patients not admitted to ICU as 3.25 and 2.95 g/dl for the patients admitted to ICU. Similarly, we found the median serum albumin level to be 3.08 g/dl for all hospitalized COVID-19 patients and a value of 2.74 g/dl for patients who died.

Considering clinical data, this study reaches the same conclusion as that of other studies, where patients’ age and chronic disease conditions are critical in predicting COVID-19 prognostics, especially in mortality [6, 62, 63].

### Limitations

This study highlights the value of ML algorithms in prognostic predictions. However, this study has several limitations. First, the study collected data from a single healthcare center. Involving more datasets from multiple centers could have resulted in more reliable prediction models. Similarly, studies revealed that COVID-19 might have different effects on diverse racial and ethnic groups. Hence, collecting data from a single country might limit the generalization of the findings. Second, the models used in this study might involve bias due to unclear reporting, assumptions made to manage missing values, and model overfitting. Third, only an internal validation study was conducted. The inclusion of external validation could have allowed more insights into the generalizability of the prediction models [64]. Indeed, the scaled brier scores also indicated low to poor clinical use with the results obtained in Models 1, 2 and 3, without conducting external validation. However, the integrated use of two datasets revealed promising results in Models 4, 5 and 6. Fourth, the study involved data from patients across a wide age range with low density at certain ages. This limits modes’ learning ability. Fifth, there was a naturalistic imbalance between labels in each dataset, patients in ICU, intubated, or died. Sixth, this study used a relatively small sampling data size, especially in Models 4, 5 and 6.

Although ML algorithms have some common pitfalls [64], they can still complement the skills of healthcare professionals in patient prioritization, prognostic decision-making, and resource management. Despite all limitations, this study used a considerably high number of COVID-19 patient data containing thirty-seven features drawn from patient demographics, clinical data, and blood test results. A healthcare professional assisted in developing reliable assumptions, and the study reported high AUROC values for the performance of each model.

## Conclusion

This study used eight supervised ML algorithms to predict the need for intensive care, intubation, and the risk of mortality for COVID-19 patients. By estimating all three prognostic predictions for individual patients, this study provides a different approach to addressing the multi-label problems in ML.

This study demonstrated the promising value of ML in COVID-19 prognostic predictions. The findings indicate that tree-based classifier algorithms perform best, although all algorithms obtained an AUROC value of over 80 percent. Future studies might develop a hybrid approach using tree-based algorithms, which could be applied in broader healthcare settings.

## Supplementary Information

Below is the link to the electronic supplementary material.

Supplementary file1 (DOCX 802 KB)
