# Supplementary material for: A comparison of machine learning algorithms in predicting COVID-19 prognostics
Source: Intern Emerg Med. 2022 Sep 18;18(1):229–39. doi: 10.1007/s11739-022-03101-x (PMC9483274; doi:10.1007/s11739-022-03101-x)
Supplement: Supplementary file 1 — Supplementary file1 (DOCX 802 KB) [file 11739_2022_3101_MOESM1_ESM.docx]

**Supplementary Information for**

A comparison of machine learning algorithms in predicting COVID-19 prognostics

**Supplemental Figures**

**Supplementary Figure S1.** Feature importance for the need for intensive care (Model 1). XGBoost feature importance was used to calculate the significance of each feature on the prediction model. Features having 0 value were discarded from the Figure.

**Supplementary Figure S2.** Feature importance for the need for a mechanical ventilator (Model 2). XGBoost feature importance was used to calculate the significance of each feature on the prediction model. Features having 0 value were discarded from the Figure.

**Supplementary Figure S3.** Feature importance for the risk of mortality (Model 3). XGBoost feature importance was used to calculate the significance of each feature on the prediction model. Features having 0 value were discarded from the Figure.

**Supplementary Figure S4.** Feature importance for the need for intensive care (Model 4). XGBoost feature importance was used to calculate the significance of each feature on the prediction model. Features having 0 value were discarded from the Figure.

**Supplementary Figure S5.** Feature importance for the need for a mechanical ventilator (Model 5). XGBoost feature importance was used to calculate the significance of each feature on the prediction model. Features having 0 value were discarded from the Figure.

**Supplementary Figure S6.** Feature importance for the risk of mortality (Model 6). XGBoost feature importance was used to calculate the significance of each feature on the prediction model. Features having 0 value were discarded from the Figure.

**Supplemental Tables**

**Supplementary Table S1**. Descriptive statistics. Patient demographics and clinical dataset were used to develop Model 1, 2 and 3.

| Features | Description | ICU  (n=4549) | Intubated  (n=2850) | Mortality  (n=2997) | Total  (n=11712) |
| --- | --- | --- | --- | --- | --- |
| Demographics Features | | | | | |
| Age (years) | Median [IQRs] | 68  [57, 75] | 68  [59, 76] | 72  [63, 79] | 65  [54, 75] |
| Gender  Female  Male | Number (%) | 1718 (37.8%)  2831 (62.2%) | 1054 (37%)  1796 (63%) | 1138 (38%)  1859 (62%) | 5112 (43.6%)  6600 (56.4%) |
| Clinical Features | | | | | |
| Temperature (^o^C) | Median [IQRs] | 36.5  [36.4, 36.7] | 36.5  [36.4, 36.67] | 36.5  [36.4, 36.7] | 36.5  [36.3, 36.7] |
| Heart rate (bmp) | Median [IQRs] | 81 [70, 94] | 84 [69, 98] | 84 [71, 98] | 80 [70, 89] |
| Oxygen saturation (%) | Median [IQRs] | 95 [91, 97] | 94 [90, 97] | 94 [90, 97] | 95 [93, 97] |
| Blood pressure (mmHg)  Systolic  Diastolic | Median [IQRs] | 123 [110, 138]  70 [59, 80] | 124 [110, 140]  61 [54, 70] | 122 [110, 139]  64 [55, 76] | 121[110, 134]  74 [64, 84] |
| Pupils  Isochoric (0)  Miotic (1)  Mydriatic (2) | Number (%) | 4463 (98.11%)  84 (1.85%)  2 (0.04%) | 2752 (96.56%)  96 (3.37%)  2 (0.07%) | 2914 (97.23%)  81 (2.7%)  2 (0.07%) | 11606 (99%)  104 (0.9%)  2 (0.1%) |
| Consciousness  Awake  Verbal or Pain  Unresponsive | Number (%) | 2762 (60.7%)  964 (21.2%)  823 (18.1%) | 761 (26.7%)  1190 (41.8%)  899 (31.5%) | 1270 (42.4%)  964 (32.2%)  763 (25.4%) | 9440 (80.6%)  1196 (10.2%)  1076 (9.2%) |
| General condition  Critical  Severe  Ordinary  Mild  Asymptomatic | Number (%) | 828 (18.2%)  952 (20.9%)  1041 (22.9%)  634 (13.9%)  1094 (24.1%) | 940 (33%)  1073 (37.6%)  673 (23.6%)  50 (1.8%)  114 (4%) | 808 (26.96%)  934 (31.16%)  515 (21.6%)  279 (8.2%)  461 (12.6%) | 1046 (8.9%)  1156 (9.9%)  2079 (17.8%)  1868 (15.9%)  5563 (47.5%) |
| Diuresis  Yes  No  Inadequate | Number (%) | 4246 (93.3%)  303 (6.7%) | 2508 (88%)  342 (12%) | 2677 (89.3%)  320 (10.7%) | 11343 (96.8%)  369 (3.2%) |
| Cardiovascular diseases | Number (%) | 1448 (31.8%) | 841 (29.5%) | 1074 (35.8%) | 2867 (24.5%) |
| Hypertension | Number (%) | 2307 (50.7%) | 1415 (49.6%) | 1426 (47.6%) | 4640 (39.6%) |
| Diabetes mellitus | Number (%) | 1620 (35.6%) | 977 (34.3%) | 1027 (34.3%) | 3208 (27.4%) |
| Neurological diseases | Number (%) | 438 (9.6%) | 289 (10.1%) | 296 (9.9%) | 814 (6.95%) |
| Respiratory diseases | Number (%) | 710 (15.6%) | 363 (12.7%) | 461 (15.4%) | 1432 (12.2%) |
| Benign prostate hyperplasia | Number (%) | 277 (6.1%) | 143 (5%) | 204 (6.8%) | 415 (3.54%) |
| Chronic renal failure | Number (%) | 244 (5.4%) | 141 (4.9%) | 285 (9.5%) | 726 (6.2%) |
| Hepatitis C | Number (%) | 29 (0.6%) | 16 (0.56%) | 0 | 29 (0.025%) |
| Cancer | Number (%) | 143 (3.1%) | 96 (3.4%) | 100 (3.3%) | 400 (3.4%) |

**Supplementary Table S2.** Descriptive statistics. Patient demographics, clinical data and blood test results were used to develop Model 4, 5 and 6. Values are represented as median [interquartile] or number (%).

| Features | ICU  (n=293) | Intubated  (n=197) | Mortality  (n=183) | Total  (n=602) |
| --- | --- | --- | --- | --- |
| Demographic Features | | | | |
| Age (years) | 66 [61, 73] | 66 [61, 73] | 66 [63, 74] | 66 [58.25, 72] |
| Gender  Female  Male | 74 (25.3%)  219 (74.7%) | 41 (20.8%)  156 (79.2%) | 57 (31.1%)  126 (68.9%) | 220 (35.5%)  382 (63.5%) |
| Clinical Features | | | | |
| Temperature (^o^C) | 36.5 [36.4, 36.8] | 36.5 [36.4, 36.7] | 36.5 [36.4, 36.85] | 36.5 [36.3, 36.7] |
| Heart rate (bmp) | 85 [74, 97] | 87 [75, 101] | 85 [74, 98.5] | 81 [72, 92] |
| Oxygen saturation (%) | 95 [91, 96] | 94 [90, 96] | 94 [90, 96] | 95 [92, 97] |
| Blood pressure (mmHg)  Systolic  Diastolic | 121 [108, 137]  68 [59, 79] | 120 [107, 140]  63 [55, 70] | 118 [105, 137]  64 [54.5, 71] | 121 [110, 135]  70 [63, 82] |
| Pupils  Isochoric  Miotic  Mydriatic | 283 (99.7%)  10 (0.03%)  0 | 196 (99.5%)  1 (0.05%)  0 | 182 (99.5%)  1 (0.05%)  0 | 578 (96%)  24 (4%)  0 |
| Consciousness  Awake  Verbal or Pain  Unresponsive | 165 (56.3%)  67 (22.9%)  61 (20.8%) | 57 (28.9%)  75 (38.1%)  65 (33%) | 83 (45.4%)  54 (29.5%)  46 (25.1%) | 447 (74.3%)  80 (13.3%)  75 (12.4%) |
| General condition  Critical  Severe  Ordinary  Mild  Asymptomatic | 68 (23.2%)  57 (19.5%)  52 (17.7%)  38 (13%)  78 (26.6%) | 67 (34.01%)  67 (34.01%)  39 (19.8%)  5 (2.54%)  19 (9.64%) | 51 (27.9%)  51 (27.9%)  29 (15.8%)  15 (8.2%)  37 (20.2%) | 81 (13.5%)  70 (11.6%)  91 (15.1%)  110 (18.3%)  250 (41.5%) |
| Diuresis  Yes  No | 270 (92.2%)  23 (7.8%) | 174 (88.3%)  23 (11.7%) | 167 (91.26%)  16 (8.74%) | 578 (96%)  24 (4%) |
| Cardiovascular diseases | 78 (26.6%) | 47 (23.9%) | 63 (34.4%) | 139 (23%) |
| Hypertension | 145 (49.5%) | 105 (53.3%) | 94 (51.4%) | 279 (46.3%) |
| Diabetes mellitus | 83 (28.3%) | 57 (28.9%) | 53 (28.96%) | 193 (32%) |
| Neurological diseases | 46 (15.7%) | 48 (24.4%) | 20 (10.93%) | 75 (12.5%) |
| Respiratory diseases | 52 (17.7%) | 34 (17.3%) | 52 (28.42%) | 94 (15.6%) |
| Benign prostate hyperplasia | 31 (10.6%) | 25 (12.7%) | 31 (16.94%) | 31 (5.1%) |
| Chronic renal failure | 15 (5.1%) | 11 (0.06%) | 18 (9.84%) | 58 (9.6%) |
| Hepatitis C | 8 (2.7%) | 4 (0.02%) | 0 | 8 (1.3%) |
| Cancer | 0 | 2 (0.01%) | 0 | 34 (5.6%) |
| Blood Test Features | | | | |
| Alanine aminotransferase (U/L) (serum) | 44[20, 61] | 45 [22, 61] | 46.5 [18.5, 67.88] | 30.5 [16, 52.8] |
| Aspartate aminotransferase (U/L) (serum) | 35 [22, 56] | 40.6 [27, 62] | 40 [24, 62.5] | 27.5 [16, 45] |
| White blood cell count (10^3/uL) | 9.6 [6.3, 13.9] | 11 [7.9, 15.3] | 9.5 [5.35, 15.7] | 8.2 [5.5, 11.9] |
| Platelet count (10^3/uL) | 200 [110, 291] | 166 [102, 270] | 136.17 [83, 223.5] | 200 [119, 280.8] |
| Mean platelet volume (fL) | 10.5 [9.5, 11.8] | 11.4 [9.9, 12.5] | 11.3 [10, 12.6] | 10.7 [9.8, 11.85] |
| Eosinophil count (10^3/uL) | 0 [0, 0.02] | 0 [0, 0.01] | 0 [0, 0.0009] | 0.003 [0, 0.03] |
| Neutrophil count (10^3/uL) | 8.69 [4.95, 12.23] | 9.89 [6.79, 14.05] | 8.69 [4.6, 14.46] | 6.76 [4.2, 10.54] |
| Lymphocyte count (10^3/uL) | 0.6 [0.3, 0.9] | 0.5 [0.2, 0.8] | 0.3 [0.18, 0.6] | 0.7 [0.1, 1.1] |
| Basophil count (10^3/uL) | 0 [0, 0.01] | 0 [0, 0.01] | 0 [0, 0.0067] | 0.005 [0, 0.01] |
| Lactate dehydrogenase (U/L) (serum) | 387 [293.8, 478] | 437.4 [331, 530] | 422 [325, 516.5] | 343 [255.3, 432] |
| Glucose ﻿(mg/dL) (serum) | 151 [121, 194] | 168 [128, 218] | 169 [138, 215.77] | 154 [117, 206.5] |
| Urea (mg/dL) (serum) | 68 [40, 107] | 77 [43, 127] | 84.3 [50.5, 124.5] | 55 [37, 93] |
| Albumin ﻿(g/L) (serum) | 28.4 [26.5, 30.8] | 28.2 [25.4, 30.8] | 27.4 [25.45, 29.95] | 30.85 [27, 34] |
| Sodium ﻿(mmol/L) (serum) | 139 [134, 141.15] | 141 [137, 147] | 142 [137, 147] | 137 [134, 141] |
| Potassium ﻿(mmol/L) (serum) | 3.9 [3.6, 4.4] | 3.9 [3.5, 4.3] | 3.9 [3.6, 4.2] | 4.04 [3.7, 4.5] |
| Magnesium (mg/dL) (serum) | 2.08 [1.83, 2.22] | 2.1 [1.83, 2.29] | 2.11 [1.9, 2.3] | 2.03 [1.8, 2.19] |
| C-reactive protein ﻿(mg/L) (serum) | 47.42 [19.08, 89.17] | 47.42 [20.7, 88.81] | 58.79 [23.79, 98.93] | 32.4 [11.9, 70] |
| Creatinine (serum) | 0.82 [0.62, 1.16] | 0.85 [0.61, 1.41] | 0.96 [0.67, 1.6] | 0.88 [0.67, 1.2] |

**Supplementary Table S3.** Correlation matrix for the features of demographics and clinical data. Pearson correlation values were represented. Values are between +1 (represents positive correlation) and -1 (represents negative correlation). Values close to 1 or -1 means strong correlations, whereas values close to 0 represent weak correlations.


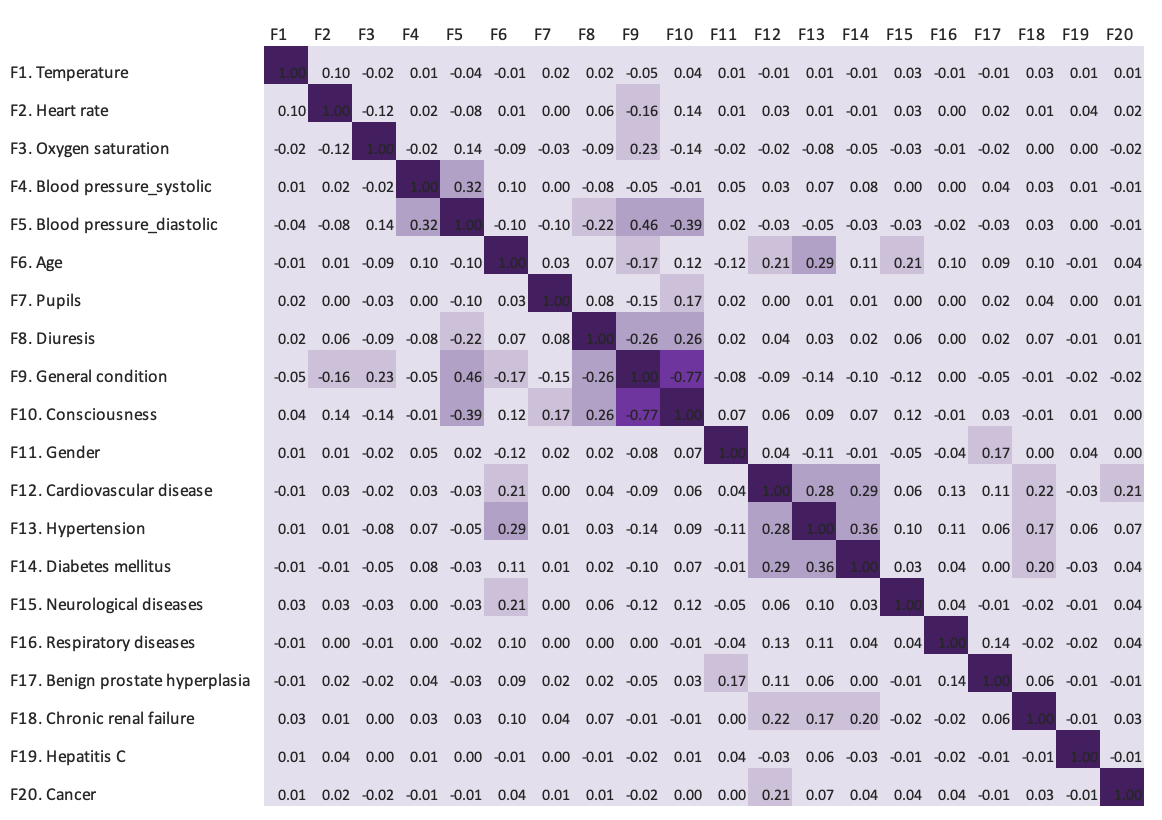


**Supplementary Table S4.** Correlation matrix for the features of demographics, clinical data and blood test results. Pearson correlation values were represented. Values are between +1 (represents positive correlation) and -1 (represents negative correlation). Values close to 1 or -1 means strong correlations, whereas values close to 0 represent weak correlations.


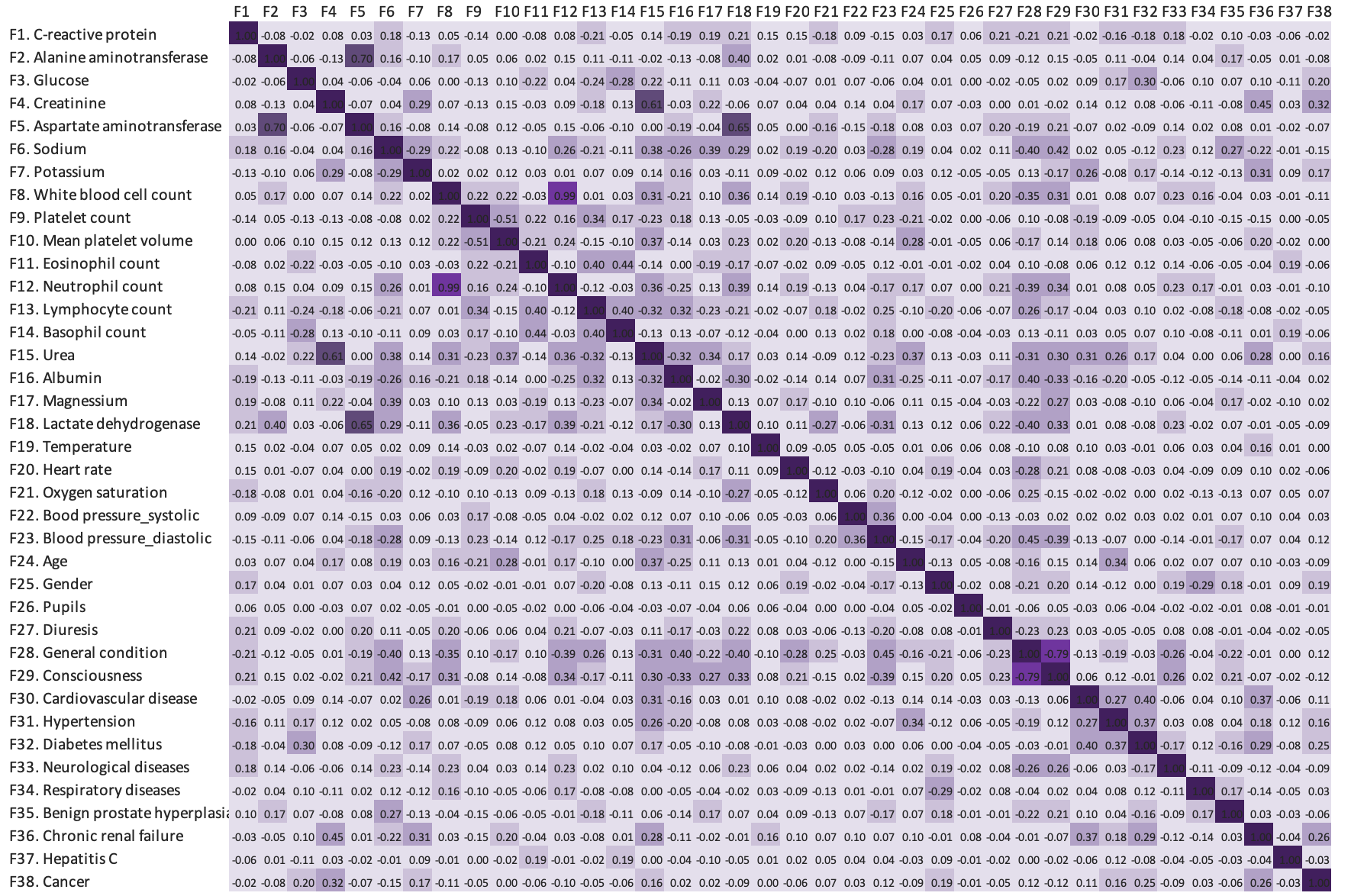


**Supplementary Table S5.** Tuning hyperparameters for the study.

| Extreme Gradient Boosting | "max_depth": [2, 3, 4, 5, 6, 7],  'learning_rate': [0.005, 0.015, 0.025, 0.035, 0.045, 0.055, 0.065, 0.075, 0.085,0.095, 0.105, 0.115, 0.125, 0.135, 0.145, 0.155, 0.165, 0.175,0.185, 0.195, 0.205, 0.215, 0.225, 0.235, 0.245, 0.255, 0.265,0.275, 0.285, 0.295]  "n_estimators": [ 25,  50,  75, 100, 125, 150, 175, 200, 225, 250, 275]     "gamma": [0.5 , 0.55, 0.6 , 0.65, 0.7 , 0.75, 0.8 , 0.85, 0.9 , 0.95]) "reg_alpha": [0.5 , 0.75, 1.  , 1.25] |
| --- | --- |
| CatBoost Classifier | "depth": [3, 1, 2, 6, 4, 5, 7, 8, 9, 10], "iterations": [250, 100, 500, 1000], "learning_rate": [0.03, 0.001, 0.01, 0.1, 0.2, 0.3] "l2_leaf_reg": [3, 1, 5, 10, 100], "border_count": [32, 5, 10, 20, 50, 100, 200] |
| Extra Tree Classifier | "n_estimators": *[10, 20, 30, 40, 50, 60, 70, 80, 90, 100]*  "criterion": ["gini", "entropy"],  "max_depth": [10, 20, 30, 40, 50, 60, 70, 80, 90, 100, 110]  "min_samples_split": [2, 5, 7, 9, 10],  "min_samples_leaf": [1, 2, 4],  "max_features": ["auto", "sqrt", "log2"] |
| Random Forest Classifier | "n_estimators": [10, 20, 30, 40, 50, 60, 70, 80, 90, 100],  "criterion": ["gini", "entropy"],  "max_depth": [10, 20, 30, 40, 50, 60, 70, 80, 90, 100, 110]    "min_samples_split": [2, 5, 7, 9, 10],  "min_samples_leaf": [1, 2, 4],  "max_features": ["auto", "sqrt", "log2"] |
| MLP Classifier | "activation": ["relu", "tanh"]  "solver": ["adam", "lbfgs", "sgd"  "alpha": hp.choice("x_alpha", 10.0 ** -np.arange(1, 7)),  "max_iter": [100, 200, 300, 400, 500, 600, 700, 800, 900]  "hidden_layer_sizes": [ 5,  6,  7,  8,  9, 10, 11]  "batch_size": [ 5, 10, 15, 20, 25]   "learning_rate_init": [0.01, 0.03, 0.1] |
| Logistic Regression | "C": *[1,5,10,25,50,100],* "penalty": ["l1", "l2"], |
| Support Vector Machine | 'loss': ["hinge", "log", "squared_hinge", "modified_huber"]   'alpha': [0.0001, 0.001, 0.01, 0.1]   'penalty': ["l2", "l1", "elasticnet", "none"       'eta0': hp.choice("x_eta0", [0.001, 0.01, 0.05, 0.1, 0.2, 0.3, 0.4, 0.5]   'learning_rate': ["constant", "optimal", "invscaling", "adaptive" |
| K Neighbours Classifier | "n_neighbors": [ 1,  2,  3,  4,  5,  6,  7,  8,  9, 10, 11, 12, 13, 14, 15, 16, 17, 18, 19, 20, 21, 22, 23, 24, 25, 26, 27, 28, 29, 30, 31, 32, 33, 34, 35, 36, 37, 38, 39, 40, 41, 42, 43, 44, 45, 46, 47, 48, 49, 50]   "weights": ["uniform", "distance"]   "metric": ["euclidean", "manhattan"] |
